# Insights and Strategies to Revive Brachytherapy Using Social Media: A Google Trends Analysis

**DOI:** 10.7759/cureus.25664

**Published:** 2022-06-04

**Authors:** Kaidi Wang, Gary Lewis

**Affiliations:** 1 Department of Radiation Oncology, University of Arkansas for Medical Sciences, Little Rock, USA

**Keywords:** public health, google trends, cancer, brachytherapy, social media

## Abstract

Introduction: The utilization and public awareness of brachytherapy are both declining. Social media has an increasing presence in health promotions. As regards cancer care, social media has been successfully used as a platform for information dissemination, psychosocial support, and patient engagement and empowerment.

Methods and materials: Using Google Trends (Google LLC, Mountain View, CA, USA), we analyzed the impacts on the public interest of three brachytherapy-related social media campaigns/publicity events and compared and contrasted them with three other campaigns/publicity events. We used descriptive statistics (mean ± standard deviation (SD)) to describe the search results, independent t-tests to compare means before and after campaigns/announcements for short-term effects, and one-way ANOVA (or Kruskal-Wallis test when appropriate) to compare mean values across distinct time periods for long-term effects.

Results: We identified three major types of social media campaigns/events: those that have a short-term impact but little long-term impact, those that have both short-term and long-term impacts, and those with little short-term or long-term impact. We examined campaigns with significant and lasting impacts and noticed that they tend to be celebrity-related/celebrity-endorsed, focused on sharing personal experiences, and occur with regular frequency.

Conclusions: To increase public awareness of brachytherapy, the American Brachytherapy Society (ABS) can consider tie-ins with events and people with high search traffic (such as Breast Cancer Awareness Month), having celebrities/influencers who were treated with brachytherapy to provide testimonials, encouraging patient engagement and sharing of their experiences with brachytherapy on social media, and setting up recurring brachytherapy publicity events.

## Introduction

Unlike external radiation beams that are generated by machines and delivered to the patient at a distance, brachytherapy consists of placing sealed radioactive sources close to or in contact with the area of treatment of the patient [1]. The sources chosen for brachytherapy can be categorized into high-dose rate (HDR) defined as greater than 12 Gy per hour and lose-dose rate (LDR) defined as less than 2 Gy per hour [2]. They are different from those of external beam therapy in that both HDR and LDR sources have rapid dose falloffs, typically <10% of dose delivered to tissue >4 cm away from the source [2]. The rationale for brachytherapy is to deliver high doses with rapid dose falloffs over a short amount of time, thereby sparing nearby normal organs as much as possible. Brachytherapy has been commonly utilized in the treatment of breast cancer, prostate cancer, gynecological cancers such as cervical cancer and endometrial cancer, ocular cancer, and skin cancer [3]. For the definitive treatment of locally advanced cervical cancer, brachytherapy is an essential component of curative treatment; omission of brachytherapy results in decreased survival [4]. Unfortunately, in recent years, the utilization and public awareness of brachytherapy are both declining [5,6]. The reasons are multifold, such as high costs to start and maintain brachytherapy programs, increase in the technical sophistication of and reimbursement for external beam radiation therapy, reduced referrals from surgeons due to advances in surgical equipment, and limited brachytherapy experience for physicians and residents [7,8]. Social media has an increasing presence in health promotions [9,10]. As regards cancer care, social media has been successfully used as a platform for information dissemination, psychosocial support, and patient engagement and empowerment [11,12]. Using Google Trends (Google LLC, Mountain View, CA, USA), we analyzed the impacts on the public interest of three brachytherapy-related social media campaigns/publicity events [13] and compared and contrasted them with three other campaigns/publicity events with varying levels of impact to uncover the traits of successful social media campaigns.

## Materials and methods

Google Trends (Google LLC, Mountain View, CA) is a search engine analytics tool that analyzes the popularity of search queries. After a user specifies a query term, time period, and location, Google Trends generates a series of search volume indices (SVIs), which are normalized search numbers scaled from 0 to 100 based on geography and time range.

We queried Google Trends using the search term “brachytherapy” within the United States during different time periods. We selected three brachytherapy-related media releases or social media campaigns, including a statement regarding HDR brachytherapy use in locally advanced cervical cancer by the American Brachytherapy Society (ABS) published on November 15, 2018, as well as “#ThisIsBrachytherapy” Twitter campaigns on July 17, 2019, and September 1, 2020. For comparison, we also queried amyotrophic lateral sclerosis (“ALS”) as a search term related to the “Ice Bucket Challenge” Twitter campaign raising awareness for ALS, “prostate cancer” after Ben Stiller announced his cancer diagnosis, and “breast cancer” after Angelina Jolie published about her prophylactic mastectomy. For each aforementioned publicity event, we searched for the SVI for 15 days before and after the media release to see short-term effects. In addition, we searched for the SVI two months before and eight months after each release (a total of 10 months) to see long-term effects. We used descriptive statistics (mean ± standard deviation (SD)) to describe the search results, independent t-tests to compare means before and after campaigns/announcements for short-term effects, and one-way ANOVA (or Kruskal-Wallis test when appropriate) to compare mean values across distinct time periods for long-term effects. Statistical analyses were performed using SPSS version 25 (IBM Corp., Armonk, NY, USA); significance was considered at p < 0.05.

## Results

**Figure 1:**
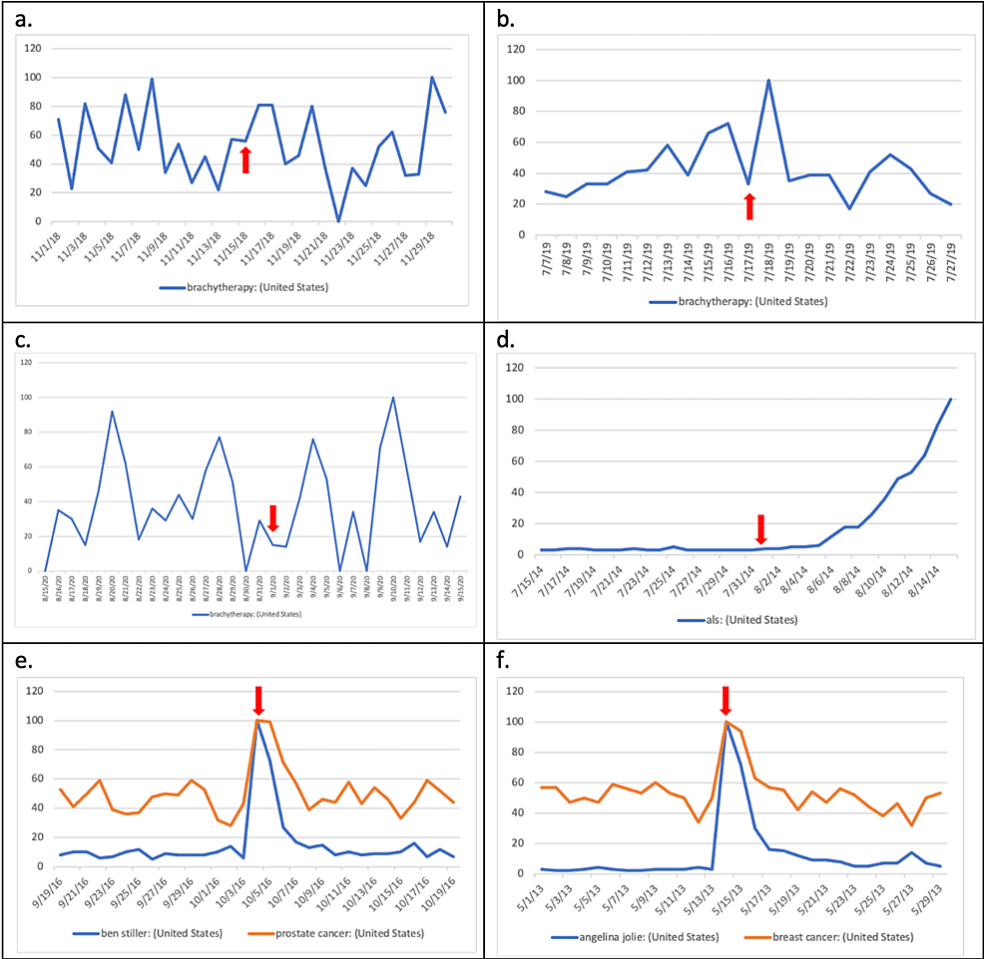
Line graphs of search volume indices shortly before and after each of the following events: a) ABS, b) first “ThisIsBrachytherapy” Twitter campaign, c) second “ThisIsBrachytherapy” Twitter campaign, d) ALS “Ice Bucket Challenge,” e) Ben Stiller announced prostate cancer diagnosis, and f) Angelina Jolie announced prophylactic double mastectomy surgery. Red arrows represent the time of occurrence of each event.

**Figure 2:**
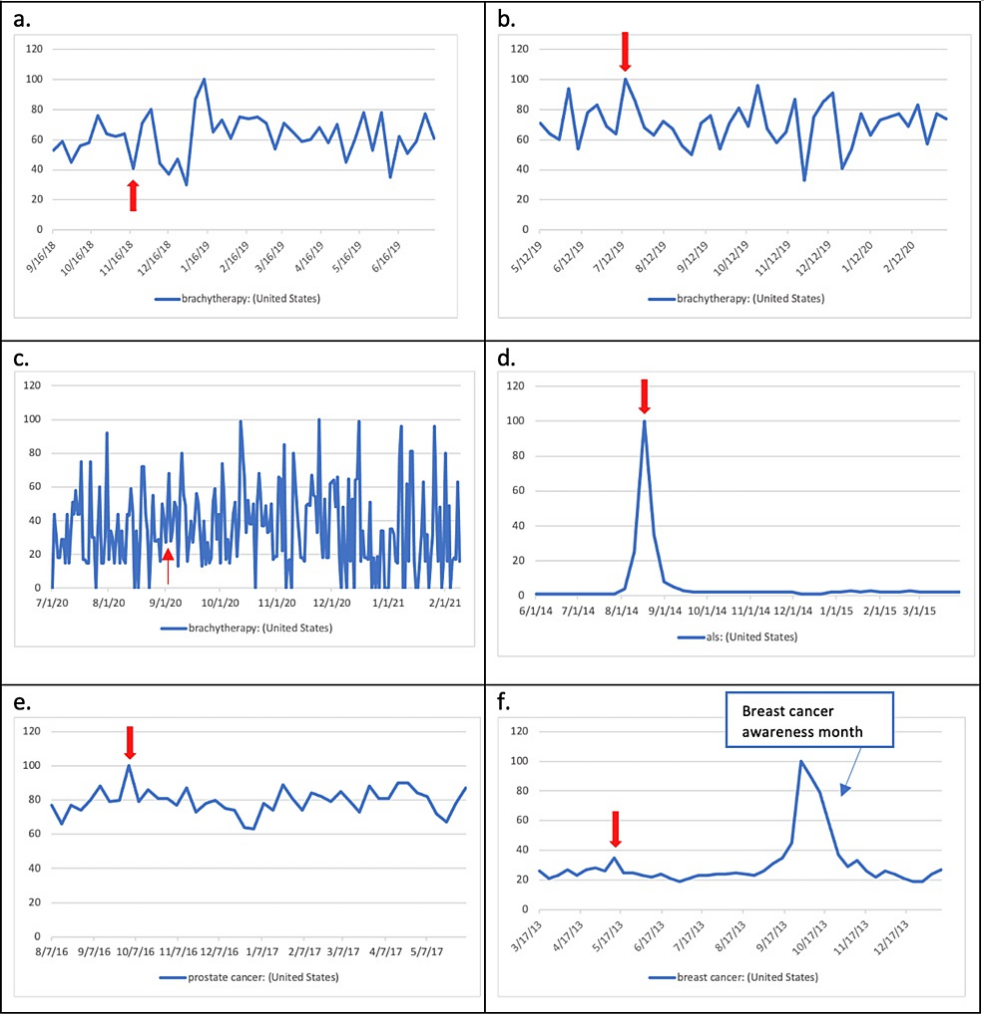
Line graphs of search volume indices two months before and eight months after each of the following events: a) ABS, b) first “ThisIsBrachytherapy” Twitter campaign, c) second “ThisIsBrachytherapy” Twitter campaign, d) ALS “Ice Bucket Challenge,” e) Ben Stiller announced prostate cancer diagnosis, and f) Angelina Jolie announced prophylactic double mastectomy surgery. Red arrows represent the time of occurrence of each event.

We first examined the short-term impact on SVI of the ABS statement using an independent t-test (Table 1). Despite a moderate increase in Internet search volume in the following two days (Figure 1a), the difference between the means of 15 days before statement issuing and 15 days after was not statistically significant, implying that ABS statement issuing had little short-term impact on public interest. We then examined the long-term impact on SVI using the Kruskal-Wallis test due to nonhomogeneous variances (Figure 2a). The differences among two-month intervals following the issuance were statistically insignificant (Table 2), implying that the statement issuing had little long-term effect on public interest.

**Table 1:** Short-term changes in search volume index before and after each campaign. ‡SD: standard deviation *p-values calculated using an independent t-test

Queried term	Representative campaign/event	15 days before campaign (mean (SD‡))	15 days after campaign (mean (SD))	p*
Brachytherapy	ABS announcement	53.33 (23.375)	52.13 (27.084)	0.898
	“ThisIsBrachytherapy” first Twitter campaign	38.53 (30.043)	46.00 (23.788)	0.448
	“ThisIsBrachytherapy” second Twitter campaign	38.35 (24.749)	38.07 (29.380)	0.976
ALS	ALS “Ice Bucket Challenge”	3.33 (0.594)	34.21 (31.222)	0.003
Ben Stiller	Ben Stiller announced prostate cancer diagnosis	9.93 (3.575)	25.75 (27.598)	0.038
Prostate cancer		42.00 (8.468)	51.81 (18.922)	0.076
Angelina Jolie	Angelina Jolie announced prophylactic double mastectomy surgery	2.85 (0.689)	20.06 (26.973)	0.022
Breast cancer		51.77 (6.870)	55.19 (18.086)	0.526

**Table 2:** Long-term changes in search volume index up to eight months after each campaign. ‡SD: standard deviation *p-values obtained from post hoc Dunnett t-test compared with “two months before campaign” #p-values obtained from one-way ANOVA (or Kruskal-Wallis test when appropriate)

Campaign/event	Two months before campaign	Two months after campaign		Four months after campaign		Six months after campaign		Eight months after campaign		p^#^
	Mean (SD‡)	Mean (SD)	p*	Mean (SD)	p	Mean (SD)	p	Mean (SD)	p	
ABS announcement	59.13 (8.983)	60.10 (23.760)	1.000	68.50 (7.746)	0.505	61.78 (7.997)	0.986	61.56 (14.475)	0.990	0.514
“ThisIsBrachytherapy” first Twitter campaign	70.75 (13.275)	70.33 (15.075)	1.000	70.78 (12.568)	1.000	67.33 (20.857)	0.968	73.13 (7.643)	0.992	0.806
“ThisIsBrachytherapy” second Twitter campaign	31.95 (20.612)	28.92 (20.776)	0.803	24.70 (24.611)	0.189	24.98 (24.679)	0.249	n/a	n/a	0.248
ALS “Ice Bucket Challenge”	1 (0.000)	20.33 (32.098)	0.014	1.89 (0.333)	1.000	1.67 (0.707)	1.000	2.00 (0.000)	1.000	0.020
Prostate cancer (Ben Stiller)	77.63 (6.209)	82.44 (7.876)	0.439	75.33 (8.155)	0.905	80.56 (4.927)	0.806	81.22 (7.855)	0.678	0.230
Breast cancer (Angelina Jolie)	25.13 (2.475)	23.89 (4.622)	0.999	24.78 (2.539)	1.000	56.22 (26.939)	0.000	23.11 (3.018)	0.991	0.000

With the first “#ThisIsBrachytherapy” Twitter campaign on July 17, 2019, a sharp increase in Internet search volume the day following the campaign was seen, which quickly returned to baseline the next day (Figure 1b). When we tested short-term impact using an independent t-test, however, the result was statistically insignificant (Table 1). Using one-way ANOVA, we see little long-term impact at two-month intervals up to eight months after the campaign (Table 2). Similarly, we analyzed SVI for the second Twitter campaign on September 1, 2020, and the results showed no statistical significance (Tables 1, 2).

Following Ben Stiller’s announcement about his prostate cancer diagnosis and Angelina Jolie’s article on having a prophylactic double mastectomy, we saw noticeable peaks in SVIs for the terms “Ben Stiller,” “prostate cancer,” “Angelina Jolie,” and “breast cancer” (Figure 1e, 1f). We analyzed short-term impacts again using independent t-tests. The search terms “Ben Stiller” and “Angelina Jolie” yielded a statistically significant increase (p = 0.038 and p = 0.022, respectively); the search terms “prostate cancer” and “breast cancer” showed an increase in SVI that were not statistically significant. Given that prostate cancer and breast cancer are the two most prevalent cancers [14], we think that their high search frequencies at baseline rendered such increases statistically insignificant. When analyzed for long-term impact using one-way ANOVA, neither produced any long-term impacts (Table 2).

**Figure 3:**
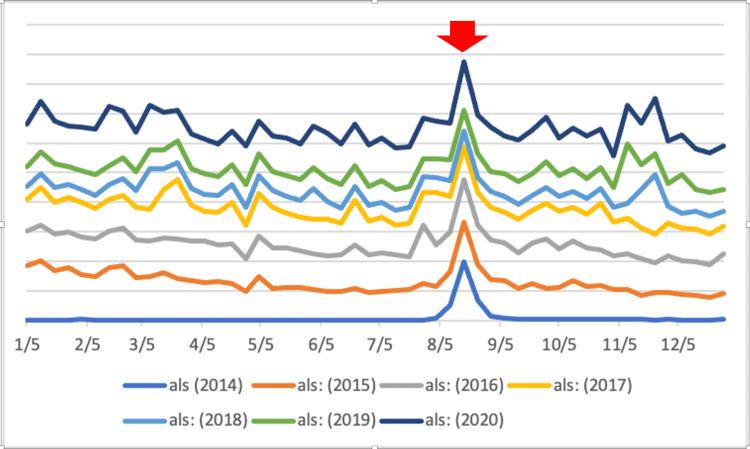
Stacked line graph showing a recurrent pattern of increase in the search frequency of “ALS” in August of every year since 2014, corresponding to the annual “Ice Bucket Challenge” raising awareness for ALS.

The ALS “Ice Bucket Challenge” clearly resulted in increases in SVI for “ALS” both in the short term (Figure 1d) and long term (Figure 2d). It also resulted in statistically significant increases in the short term (p = 0.003) and up to two months after the first campaign (p = 0.014). Moreover, when we examined the SVI on a yearly basis (Figure 3), we saw a recurrent pattern of increase in search frequency of “ALS” in August of every year since 2014, corresponding to the annual “Ice Bucket Challenge” campaign.

## Discussion

Google Trends helped us identify three major types of social media campaigns/events: those that have a short-term impact but little long-term impact (i.e., Ben Stiller and Angelina Jolie’s respective announcements), those that have both short-term and long-term impacts (i.e., the “Ice Bucket Challenge”), and those with little short-term or long-term impact (i.e., the three brachytherapy campaigns). Google Trends may be less sensitive to detect changes in commonly searched words, but it is very sensitive to detect a relatively obscure event that goes “viral,” aka gathering a massive amount of public attention within a short amount of time.

Examining campaigns with significant and lasting impacts such as the Angelina Jolie’s editorial and ALS “Ice Bucket Challenge,” we noticed several features that they had in common: they tend to be celebrity-related/celebrity-endorsed, focused on sharing personal experiences, and occur with regular frequency (such as an annual awareness month).

The impact of Angelina Jolie’s editorial on breast cancer had been termed “the Angelina Jolie effect” and was the subject of several research studies. Juthe et al. noticed a dramatic increase in online information-seeking behavior immediately after the editorial [15]. Evans et al. noted an increase in referrals to breast cancer services in the UK [16]. Using data from a large commercial insurance claims database, Desai et al. discovered that the rates of BRCA testing increased, yet mastectomy rates remained unchanged [17]. Our study is in agreement with previous studies regarding celebrity impacts.

Not only does “who” posts matter, but also “what” gets posted on social media matters. The study of Silva et al. on Twitter and skin cancer showed that personal experiences of skin cancer were highly retweeted and liked even if posted by individuals with a small crowd of followers [18]. Similarly, Wang et al. showed that personal experience-relevant content has higher audience engagement and cancer information diffusion [19]. Moreover, Thomas et al. and Jacobs et al. have shown respectively that radiation oncology patients are open and willing to share their personal treatment experiences on social media [20,21].

“When” and “where” the information is shared also play an important role. Cohen et al. and Glynn et al. both showed in their studies that cancer awareness months are effective in increasing Internet search traffic/public interest [22,23]. In addition to the proposed annual “Brachytherapy Day” on July 17 [9], we could also utilize brachytherapy’s versatility in treating various cancers (e.g., prostate cancer, cervical cancer, and breast cancer) and tie in with their respective awareness months, thus gaining multiple publicity opportunities every year. As for the platform, Antheunis et al. discovered that patients primarily used Twitter for increasing knowledge and exchanging advice in comparison with Facebook for social support and advice, yet Facebook has the largest number of users [24].

Our research has several limitations. First, despite being a readily available, cost-effective (costs none) database, Google Trends is not a perfect mirror of search activity. Only a sample instead of the entirety of Google searches is used in Google Trends [25]. As was mentioned previously, words with high search frequencies (such as commonly searched terms or “viral” terms) are not affected as much. However, rarely searched words may be more susceptible to fluctuations secondary to sampling. According to our experience, however, although the numbers vary, the trends generally stay the same. Second, information-seeking behaviors may not necessarily translate into health-seeking behaviors [26]. To fundamentally increase the utilization of brachytherapy, other areas such as cost, reimbursement, referrals, and training need to be properly addressed as well [7,8].

## Conclusions

Our analysis categorizes social media campaigns/events into three major types of varying significance and longevity and identifies real influential social media campaigns that can be studied as potential models. To increase public awareness of brachytherapy, the ABS can consider tie-ins with events and people with high search traffic (such as Breast Cancer Awareness Month), having celebrities/influencers who were treated with brachytherapy to provide testimonials, encouraging patient engagement and sharing of their experiences with brachytherapy on social media, and setting up recurring brachytherapy publicity events.
